# Accuracy and Artistry in Anatomical Illustration of Perivascular Adipose Tissue

**DOI:** 10.3389/fphys.2017.00990

**Published:** 2017-12-04

**Authors:** Caroline M. Pond

**Affiliations:** ^1^School of Life, Health and Chemical Sciences, The Open University, Milton Keynes, United Kingdom; ^2^Department of Zoology, Oxford University, Oxford, United Kingdom

**Keywords:** Rembrandt, Leonardo da Vinci, Vesalius, anatomy tables, blood vessels, lymph nodes and vessels, site-specific properties, paracrine interactions

Research into the physiology and evolution of perivascular adipose tissue was delayed until the 1990s because the subtle influence of anatomical illustrations misled biologists.

Leonardo da Vinci's magnificent drawings, based on dissections of up to 300 human and animal cadavers, started a fashion for gross anatomy as fine art (Kemp, 2010). The many hitherto unknown details thus displayed were admired by sixteenth century cognoscenti throughout Western Europe. They presented anatomy as functional, orderly, and rational, in keeping with Renaissance values, but did not include adipose tissue around vessels or muscles (Figure 1A). Thirty years later, Vesalius studied the internal anatomy, particularly vascular systems (Vesalius, 1543), of mostly young, mostly male criminals, many executed after a period of imprisonment or other deprivation. Such subjects were probably lean, perhaps emaciated, so it is easy to understand why the spectacular and, in most respects, impressively accurate illustrations of his research followed da Vinci's habit of omitting adipose tissue (Figure 1B), promoting artistic clarity over biological accuracy.

**Figure 1:**
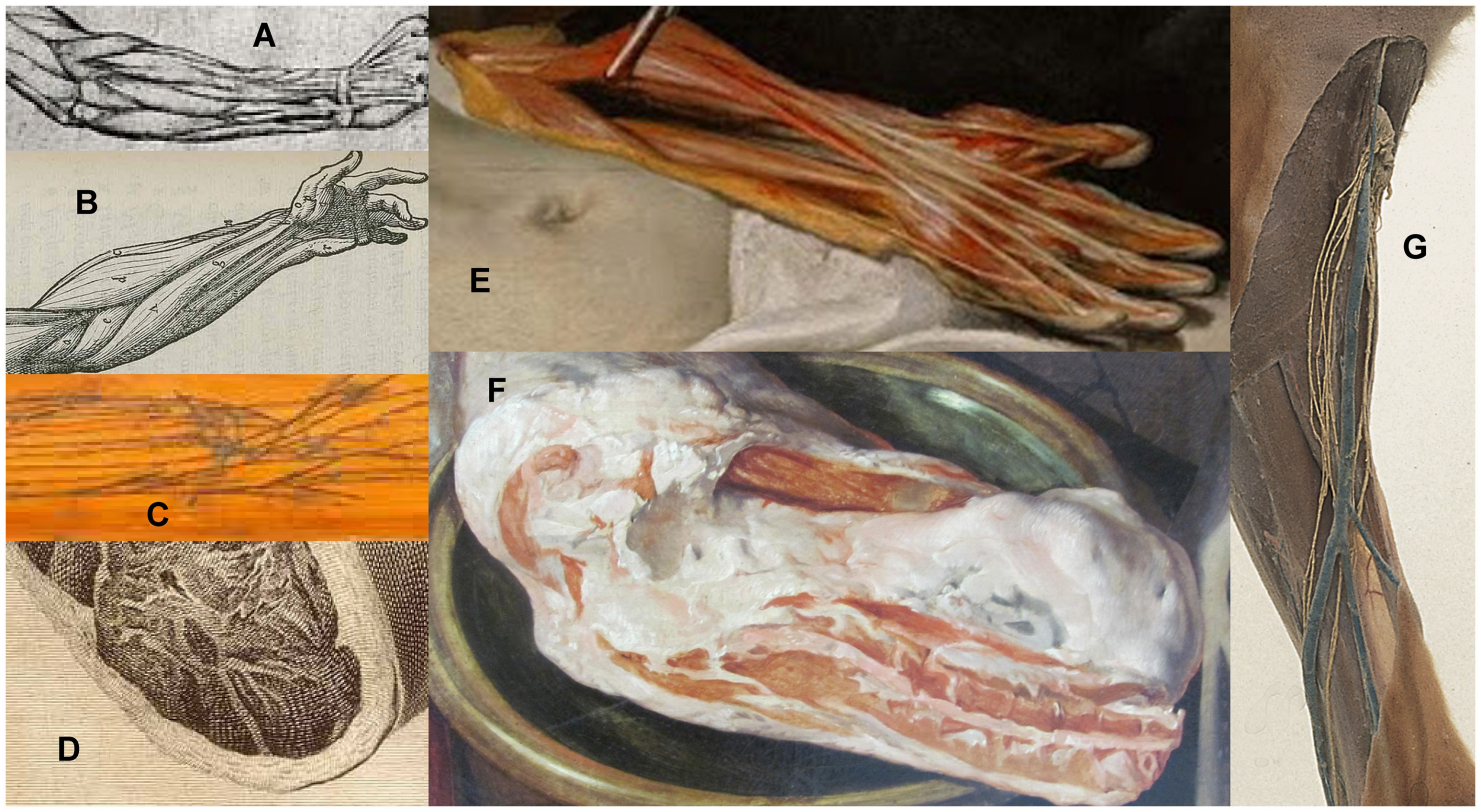
“White” adipose tissue: omitted from human forearm by **(A)** Leonardo da Vinci, c. 1510, **(B)** Vesalius 1543, **(C)** anatomy table; depicted in details from **(D)** woman's thigh in Plate VI, van Riemsdyk (Hunter, 1774), **(E)** man's arm in *The Anatomy Lesson of Dr. Nicolaes Tulp*, Rembrandt 1632, **(F)** pork in *The Four Elements: Fire*, Beuckelaer 1570. **(G)** prosection showing dog popliteal lymphatics and blood vessels (Royal College of Surgeons, $C19th).

A century after Vesalius, professional anatomists habitually improved the clarity of drawings and models by showing “important structures” dissected “free” from associated tissues. Ever more ingenious and expensive forms of anatomical illustration were developed for the amusement and erudition of wealthy intellectuals, including wooden boards to which preserved human veins, arteries, and nerves were affixed in their natural configuration (Figure 1C). By the seventeenth century, such anatomy tables were not only studied by scientists and physicians but became fashionable as curiosities (Kemp, 2010), especially in Italy. The diarist, traveler and gardener John Evelyn purchased a set in 1646 from Padua University dissectors, and brought them to London, where they were held privately for 150 years before being donated to a medical institution.

The production and display of anatomy tables had little impact on practical surgery (Kemp, 2010), which was still mainly a skill acquired through apprenticeship and experience with only a smattering of formal education. Much of the information they conveyed was irrelevant to their work that was, until anesthetics and antiseptics were invented two centuries later, mostly on limbs and superficial tissues. Opening the trunk was too dangerous, so surgeons were not concerned with the thoracic and abdominal vessels that were the most challenging to prepare and thus most impressive to artists and intellectuals.

While such anatomical illustration prevailed, a young painter struggling to gain status in Amsterdam's competitive art market depicted the tissues of the human arm as they really are (Figure 1E). In Rembrandt's 1,632 masterpiece, the “white” adipose tissue is yellow, as it always is in people (and other terrestrial vertebrates) whose diet is rich in carotenes, non-digestible plant pigments abundant in buttercup, dandelion etc. The color indicates that Adriaen Adriaenszoon's criminal career had been lucrative enough to provide plenty of carotene-rich beef, butter, and eggs, and that incarceration on a prison diet of bread and herring heads had not lasted long enough for significant depletion of his storage lipids. That artistic observation triumphed over contemporary conventions to produce a lesson in anatomical accuracy is sobering for all physicians and scientists.

Another century of advances in science and artistic realism are evident in the gynecological illustrations created by Jan van Riemsdyk for the Scottish physician and researcher, Hunter (1774). They were less coy about showing superficial and even intermuscular adipose tissue (Figure 1D) in the abdomen and thighs of their young female subjects, in whom these depots are always present, sometimes massive, but not on vessels.

Although adipose tissue on animal carcasses featured widely, and accurately, in paintings from the sixteenth century onwards (Figure 1F), human and comparative anatomists still regarded it as too inconsistent and inconsequential to be worthy of topographic, functional or evolutionary study. It was always dissected off vessels and lymph nodes in pickled prosections (Figure 1G). Illustrations in all editions of *Grey's Anatomy* from 1858 until the 1970s omit adipose tissue, though its presence is sometimes mentioned briefly in the text [Grey, 1901 (reprinted 1974)]. The artist and comparative anatomist Edwin Goodrich excluded it entirely from all his precise, detailed drawings (Goodrich, 1930). Even the modern popular presenter of anatomy, Gunther von Hagens, dissects off all adipose tissue before plastination.

These illustrators' intentions were reasonable: perivascular adipose tissue obscures anatomists' view of the vasculature. But its absence subliminally suggests that the heart, blood, and lymph vessels should be neatly sheathed in “tunics” not clad in “adventitious” adipose tissue that is at best irrelevant to vascular function, perhaps aberrant or detrimental. Microscopy using histological procedures that dissolve lipids, leaving “empty” adipocytes, further promoted the concept of “walls” around all but the smallest blood vessels and implied that all adipocytes are similar, without site-specific properties or interactions with contiguous tissues.

By the 1990s, the biochemical diversity of mammalian adipose tissues was recognized, including endocrine signaling, cytokine synthesis and reception, thermogenesis, fatty acid sorting as well as lipid storage (Pond, 2017). Studies of lymph nodes in neonatal guinea-pigs (Gyllensten, 1950) noted that they attach firmly to the surrounding adipose tissue early in development, but functional interpretation of adipocytes' anatomical relations was delayed until paracrine interactions with blood vessels (Soltis and Cassis, 1991) and lymph nodes (Pond and Mattacks, 1995, 1998) and vessels (Dixon, 2010) were demonstrated.

Paracrine interactions and diverse site-specific properties of adipose tissue were an important evolutionary advance that appeared early in mammalian evolution (Pond, 2003), facilitating the integration of competing metabolic demands of exercise, thermogenesis, efficient digestive and immune systems, and lactation (Pond, 2017). By explaining the molecular mechanisms involved, this Frontiers Research Topic should remedy the anatomists' oversight.

## Author contributions

The author confirms being the sole contributor of this work and approved it for publication.

### Conflict of interest statement

The author declares that the research was conducted in the absence of any commercial or financial relationships that could be construed as a potential conflict of interest.
